# Optically pumped subwavelength-scale metallodielectric nanopatch resonators

**DOI:** 10.1038/srep31793

**Published:** 2016-08-23

**Authors:** Kyungmok Kwon, Jong-bum You, Jaeho Shim, Youngho Jung, Kyoungsik Yu

**Affiliations:** 1 School of Electrical Engineering, KAIST, 291 Daehak-ro, Yuseong-gu, Daejeon, 34141, Korea

## Abstract

We discuss subwavelength-scale semiconductor metal-optic resonators placed on the metal substrate with various top metal plate sizes. Albeit with large optical losses, addition of metal layers converts a leaky semiconductor nano-block into a highly-confined optical cavity. Optically pumped lasing action is observed with the extended top metal layer that can significantly suppress the radiation losses. Careful investigation of self-heating effects during the optical carrier injection process shows the importance of temperature-dependent material properties in the laser rate equation model and the overall laser performances.

Integrated semiconductor-based light-emitting devices are important, indispensable building blocks for a number of emerging photonics applications, such as optical interconnects^1^,^2^,^3^, chemical sensing^4^, lighting^5^, and data storage^6^. Various optical resonator geometries have been explored to take advantages of stronger light-matter interactions at the reduced effective modal volumes as well as to shrink the overall device footprint and structural dimensions. Well-known examples of the dielectric optical resonators include one-dimensional semiconductor nanowires^7^, one-, two- or three-dimensional photonic crystals^8^,^9^,^10^,^11^, and microdisks^12^,^13^.

As the cavity size shrinks and the required lasing threshold carrier density increases, however, temperature variation during the carrier injection plays important roles in carrier dynamics and optical output power evolution^14^. The increased surface-to-volume ratios of the small cavity structures lead to enhanced non-radiative surface recombination, and the relatively low quality (Q) factors mean higher lasing threshold. Moreover, the resonator’s small heat capacity due to its small physical volume typically leads to rapid and wide temperature variation. As a result, the device temperature varies easily during the high-density carrier injection process, and the materials’ thermal properties can also vary significantly during the input pump evolution. In the conventional optical microresonator design, however, thermal issues near lasing threshold are not as important as in the subwavelength-scale resonators since heat generation from the carrier injection process does not significantly alter the overall resonator temperature due to relatively large physical volumes and heat capacities.

In this work, we discuss the design of subwavelength-scale metal-optic resonators/lasers and the influence of temperature variation on the light output intensities. To investigate thermal effects in the subwavelength-scale metal-optic resonators, we consider the temperature-dependent characteristics during the carrier injection process through the optical pumping process, and verify its effects with analytical and experimental data. Heat management issues for metal-optic resonators become more severe under the free-space optical pumping scenarios, since large portion of the pump power gets absorbed by the metal layers close to the semiconductor region and adversely affects the cavity temperature and lasing thresholds.

## Resonator Design

For rational design of metal-optic resonators, we first start our discussion by assuming a hypothetical case where a subwavelength-scale dielectric semiconductor block with the refractive index of 3.57 is surrounded by air. Such a structure can be considered as an electrically small antenna in the optical frequency regime. When its maximum dimension is comparable to a free-space wavelength, it can radiate efficiently providing a very low Q factor according to the Wheeler and McLean limit^15^,^16^. When the free-space wavelength is *λ* = 1536 nm and the dielectric semiconductor block can be enclosed by a sphere with *a* = 300 nm radius (*ka* = 2π*a*/*λ*∼1.2), the minimum radiation Q factor of such a nanoscale block from the McLean limit is 1/*k*^3^*a*^3^ + 1/*ka*∼1.4, which is in agreement with our simulation results (See Supplementary material Figures S1 and S2). The Q factor becomes worse when it is placed on a dielectric substrate because of light leakage through the bottom.

**Figure 1:**
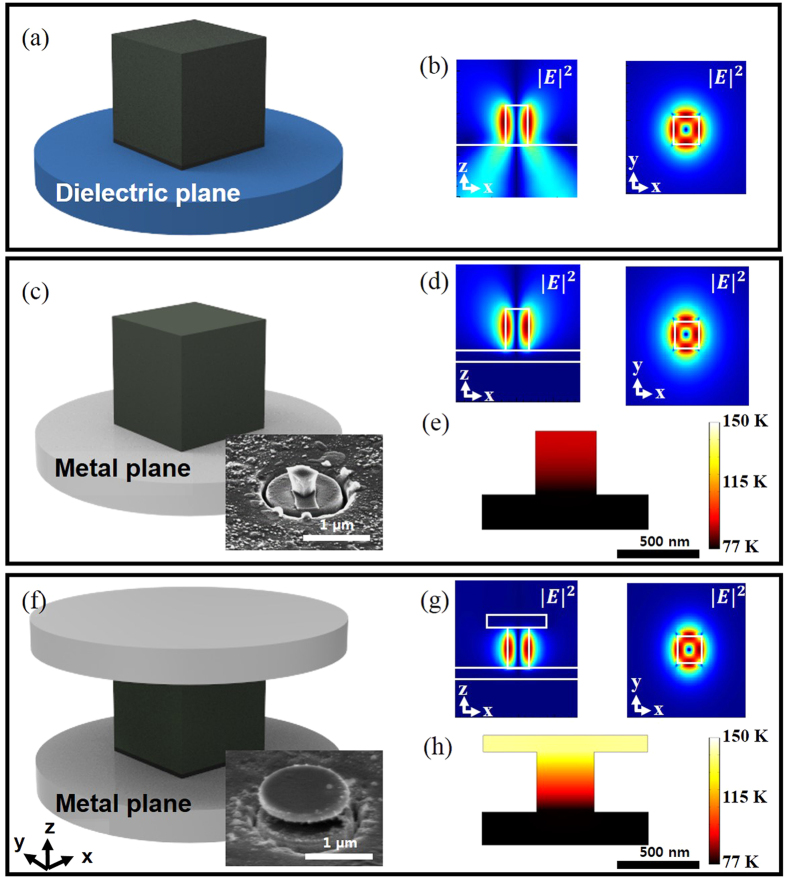
(**a**) Schematic of a subwavelength-scale semiconductor nano-block on a dielectric substrate, and (**b**) the corresponding electric field intensity profiles (side and top view) for the TE_011_–like resonant mode. The schematics of a semiconductor nano-block on a metal substrate (**c**) and an extended nanopatch resonator with a top metal layer (**f**) are shown with the corresponding scanning electron micrographs (SEMs) in the inset. The electric field intensity profiles for the TE_011_–like mode are shown in (**d,g**), respectively. The temperature profiles at a net absorbed pump power of 0.16 mW at the substrate temperature of 77 K are also shown in (**e,h**).

In contrast to the optical resonators made of purely dielectric materials, metal-optic resonators can play important roles in miniaturizing the semiconductor-based optical resonators because the presence of metal can suppress the electromagnetic radiation from the subwavelength-scale dielectric structures and thus provides strong electric field confinement within the small physical volume^17^,^18^,^19^. For example, a single isolated semiconductor nano-block on a dielectric substrate experiences severe radiation losses due to low index contrasts and the diffraction limit as shown in Fig. 1(a,b). However, placing it on a metal layer instead of the dielectric substrate can significantly reduce the unwanted radiation leakage toward the bottom, and results a much improved Q factor of ∼30 as shown in Fig. 1(c,d). Introducing an additional upper metal layer on top of the semiconductor layer allows even further optical mode confinement into the semiconductor region with a relatively high refractive index (>3), and enables the subwavelength-scale metal-optic cavity to reach a relatively high Q factor (∼400 at low temperature and ∼200 at room temperature) with a good confinement factor as illustrated in Fig. 1(f,g). In both cases, albeit with its severe absorption losses, the presence of the metal layers actually improves the light-matter interaction and the overall Purcell factor when compared with the purely dielectric cases (Q < 10).

Since the Q factors and the effective mode volumes are improved, the spontaneous emission (SE) rates from the semiconductor can also be enhanced due to the Purcell effects. In addition to the optical confinement effects from the metal layers, smaller cavity dimensions typically support fewer optical modes, and result a higher SE coupling factor (*β*) for the resonant modes. The outstanding thermal conductivities of metals can also provide superior heat dissipation and better thermal management, which are important at this small length scale^20^.

**Figure 2:**
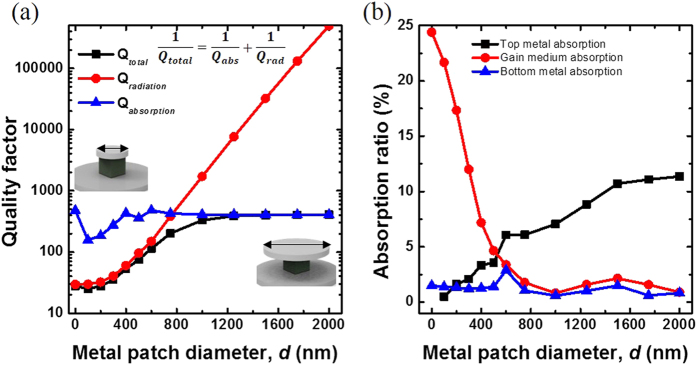
(**a**) Quality factors with respect to the top metal layer diameter, *d*. (**b**) Optical pump absorption ratio of each layer with respect to *d*. The height and width of the semiconductor nano-block are *l* = *h*=350 nm, respectively.

We verify the metal-optic semiconductor nano-block cavity designs and their operation by comparing experiments and three-dimensional finite-difference time-domain (FDTD) simulations (FDTD Solutions, Lumerical Solutions Inc.). We tentatively assume that the imaginary part of the metal permittivity would be half of the known room-temperature values since the experiments were performed at low temperature (77 K)^21^. To identify the role of the top metal layer on the metal-optic cavity performance, we numerically investigate the total cavity quality factor (*Q*_*total*_ = 1/(1/*Q*_*abs*_ + 1/*Q*_*rad*_)), where *Q*_*abs*_ and *Q*_*rad*_ represent the absorption and radiation Q factor, respectively. The height and width of the semiconductor nano-block are *h* = 350 nm and *l* = ∼350 nm, respectively, and therefore have almost a cubic shape as schematically described in Fig. 1. At this semiconductor size, degenerate TM_111_-like mode and non-degenerate TE_011_-like mode (azimuthally-polarized mode as shown in Fig. 1(b,d,g)) become dominant within the optical gain bandwidth of the semiconductor material (InGaAsP)^17^. Starting from the semiconductor cube placed on the metal substrate layer as illustrated in Fig. 1(c), we modify the metal-optic resonator structure toward Fig. 1(f) by increasing the top metal disk/patch diameter, *d*. The bottom metal substrate was assumed to be infinitely large in our FDTD simulations. As *d* increases over the semiconductor width, *l*, the radiation gets significantly suppressed and *Q*_*rad*_ improves exponentially as shown in Fig. 2(a), while the absorption losses become saturated after some point (*d* > ∼1 μm). Thus, the overall trend of the total Q factor, *Q*_*total*_, follows *Q*_*rad*_ at the initial stage (*d* < *l*), and is eventually limited by *Q*_*abs*_ when the upper metal layer becomes large enough to block the electromagnetic radiation through side opening. Although the metal layers have negative effects on the absorption losses, the overall Q factor is still much larger than the semiconductor cube structure of the similar size without any additional metal layers. The electromagnetic resonance mode is also highly localized in the semiconductor region with the effective mode volume of *V*_*eff*_ ∼1.875(*λ*/2*n*)^3^ when *d* becomes large enough. Without the top metal layer, the effective mode volume becomes larger, ∼2.162(*λ*/2*n*)^3^, resulting in a smaller Purcell factor.

In addition to the overall Q factor, the size of the top metal plate significantly affects the excitation efficiency, and therefore the temperature, of the compound semiconductor block especially when it is optically pumped from the surface normal direction. The top metal layer screens the optical pump beam, and prohibits direct excitation of the semiconductor material underneath. Figure 2(b) shows the optical excitation efficiency for the semiconductor gain region with respect to the top metal plate diameter when the input beam diameter is 2 μm. Initially without the top metal layer, a large amount of pump beam (∼24%) is absorbed into the semiconductor. However, the optical pumping efficiency significantly decreases with *d* because of absorption and reflection from the top metal layer. When *d* = 1 μm, for example, only ∼0.8% (∼7%) of the optical pump power gets absorbed at the semiconductor (top metal) layer. Our numerical simulations also indicate that, when *d* is large enough to cover the semiconductor surface (*d* > *l*), the optical pump energy is first converted to surface plasmons on the surface of the top metal layer. The surface plasmons propagate along the top metal plate and finally get absorbed by the semiconductor as explained in Figure S3. Due to the screening effects from the top metal layer, the optical pump energy transfer through direct free-space propagation is estimated to be negligible when *d* > *l*. In the proposed design, the extended top metal layer significantly improves the cavity Q factor, but has an adverse effect on the efficiency of optical pump incident from the surface normal direction.

We would like to note that the presence of the metal layers in the metallodielectric nanopatch resonators not only influences the optical pumping efficiency but also affects the overall cavity temperature, which is strongly related to the resonators’ optical performances. Examples of calculated temperature distribution profiles when the absorbed power into the semiconductor layer is 0.16 mW (close to the absorbed power at the lasing threshold in our experiments) are shown in Fig. 1(e,h), and the estimated semiconductor block temperature at its center reaches 85 and 101 K, respectively, at 10 ns after the onset of the pump irradiation when the substrate temperature is maintained at 77 K. The three-dimensional temperature profiles with respect to the various optical pumping scenarios were calculated by the finite elements method using a commercial software package (COMSOL Multiphysics, COMSOL Inc.). Under the optical pumping scenarios, the metal optic cavities with the top metal layer show worse thermal budgets when compared with the cavities without the top metal plate because of reduced optical pumping efficiencies. In the electrically driven case, however, the extended top metal plate can act as a good thermal heat sink rather than an obstacle for carrier injection.

## Results and Discussion

For experimental verification, the semiconductor nano-block structures with subwavelength-scale dimensions were fabricated on the Ag metal plane using a combination of evaporation, bonding, wet etching, and focused ion beam (FIB) patterning processes. A 350-nm-thick compound semiconductor material (InGaAsP) was epitaxially grown on an InP substrate. Plasma-enhanced chemical vapor deposition was used to grow a SiN_x_ layer (15 nm) to reduce carrier diffusion to the metal layer. Ti/Ag/Pt/Ag metal layers (the bottom metal plane in Fig. 1) were subsequently evaporated with the thicknesses of 7/300/30/2000 nm, respectively. The first silver layer was chosen for its low optical absorption losses, and slowly evaporated at a rate of <0.5 Å/s. The platinum and the second silver layer were selected for the metal diffusion barrier and the bonding layer for the subsequent metallic bonding process, respectively. After the sample was bonded to a silicon carrier wafer, the InP substrate was removed. Ti/Ag/Ti (7/80/20 nm) was evaporated to create the top metal layer. The titanium layer was used for adhesion purposes, but also introduced additional losses. The sample was then milled with FIB to pattern the initial cylindrical pillar structures. Finally, the sidewall of the epitaxial semiconductor layer was wet etched to remove the FIB-damaged sidewall surface. The fabricated device structures without and with the top metal plate are shown in the inset of Fig. 1(c,f), respectively. The diameter of the extended top metal plate is designed to be approximately 1 μm because the total Q factor is nearly saturated when *d* > 1 μm, and further increase of the top metal plate size hinders effective optical pumping.

After fabrication, the sample was placed in a cryostat vacuum chamber and cooled to 77 K. The devices are optically pumped from the surface normal direction using a 1064-nm semiconductor diode laser with a 10 ns pulse width and a 200 kHz repetition rate to allow enough time for cooling. The optical pump beam was focused by an objective lens (numerical aperture of 0.5), and the emitted light from a metal-optic resonator was collected by the same objective. The collected light signal was analyzed by a spectrometer and an InGaAs near-infrared detector array. The pump beam waist diameter was approximately 2 μm, and larger than the resonator structure.

**Figure 3:**
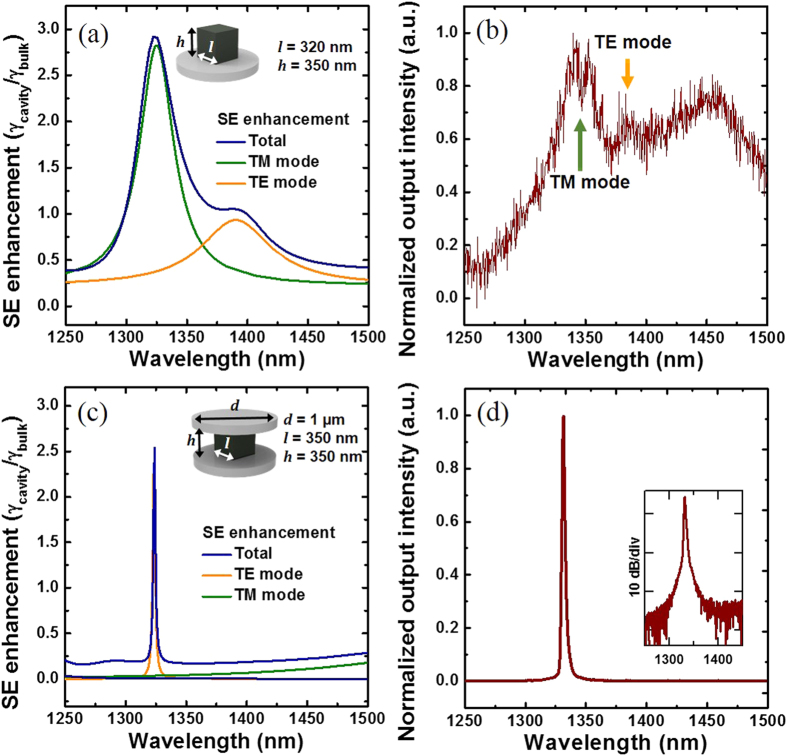
Calculated cavity-enhanced SE ratio (γ_cavity_/γ_bulk_, solid blue curves) (**a**) without and (**c**) with the top metal layer. They can be decomposed into two components: TE (solid orange) and TM mode (solid green). (**b**) Emission spectrum from a semiconductor cuboid without the top metal layer under 20 mW optical pump excitation. (**d**) Emission spectra from the extended patch resonator under the single-mode lasing condition. A log-scale plot is shown in the inset.

To investigate the effect of metal-optic resonators on SE, the enhancement of the SE rates (*γ*_cavity_/*γ*_bulk_) with the resonator structure was calculated by three-dimensional FDTD simulations and compared with experimental photoluminescence (PL) observations. When the subwavelength semiconductor block is placed on the metal substrate as described in Fig. 1(c) and the inset of Fig. 3(a), SE enhancement can be mainly decomposed to contributions from a TM and a TE mode^17^, and the TM mode emission is enhanced up to three times. This numerical calculation agrees well with the PL spectrum in Fig. 3(b) measured from the cuboid structure shown in the inset of Fig. 1(c) with *l* = 320 nm, *h* = 350 nm, and *d* = 0 μm (no top metal layer). Three major peaks in the PL spectrum correspond to the semiconductor band edge emission at ∼1450 nm as well as the metal-optic cavity resonances at ∼1340 nm (electric-dipole-like TM mode) and ∼1380 nm (magnetic-dipole-like TE mode). The SE intensity at resonance near 1340 nm is even larger than the original bulk semiconductor emission peak at 1450 nm due to the SE enhancement for the cavity mode. The measured quality factors for the resonance peak are ∼28 and ∼15 for the TM and TE mode, respectively, which are comparable to the simulation result of ∼38 and ∼19. Although such Q factors are too low to reach the lasing condition even at low temperatures for the optical gain levels attainable from typical compound semiconductor materials, this experimental result implies that the simple introduction of the metal substrate can significantly enhance the SE rate and can be employed for design of semiconductor-based light emitting structures^22^.

When the upper metal layer is introduced, the SE enhancement for the TE_011_–like cavity mode was observed as indicated in Fig. 3(c) mainly because of the improved Q factor. The measured resonance wavelength was ∼1330 nm. Due to large disparity between the metal-optic cavity resonance and SE spectrum at 77 K, however, *β* for this particular cavity design was estimated to remain relatively low over the wide range of the input pump powers and temperature. For example, when the semiconductor material is at its transparent condition and the cavity temperature is 101 K, *β* is estimated to be ∼0.017^23^,^24^. When the cavity resonance matches with the spontaneous emission peak, *β* can be improved significantly (>0.1) as discussed in previous study^17^. Figure 3(d) shows an example of optically-pumped lasing spectra from the metal-optic nanopatch resonator with the extended top metal layer (*l* = 350 nm, *h* = 350 nm, and *d* = 1 μm). The side-mode suppression well above the lasing threshold was greater than 30 dB over the semiconductor gain bandwidth, which proves the single-mode operation of the metal-optic cavity used in the experiment. The observed linewidth near the threshold (∼20 mW pump power) was ∼6.5 nm, corresponding to the cavity Q factor of ∼200. It also roughly agrees with the calculated value (∼300) from the FDTD simulations at an elevated temperature of 101 K. The measured Q factor is higher than the previously reported nanopatch resonator (*d *∼ *l*)^17^ because of better electromagnetic field confinement and reduced radiation losses from the extended top metal plate (*d* > *l*).

**Figure 4:**
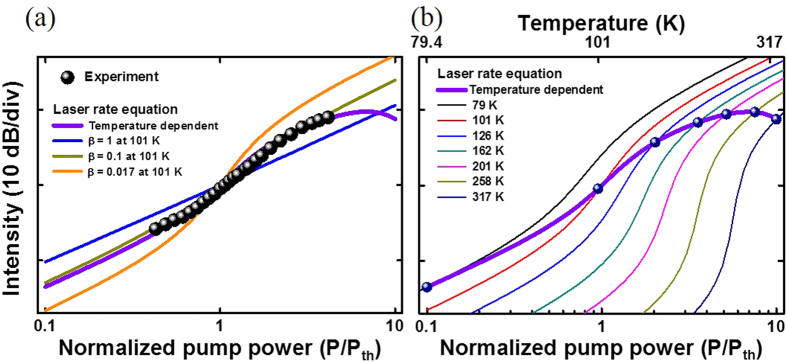
Logarithmic plots of experimental (black circles) and theoretical (solid lines) integral emission intensity. (**a**) Experimental results fit well with the temperature-dependent rate equation curve. Temperature-independent rate equation curves at 101 Kelvin for *β*=0.017, 0.1 and 1 are also shown for comparison. (**b**) The temperature-dependent output intensity curve is compared with other curves obtained from constant-temperature rate equations.

Figure 4(a) shows the laser output intensity evolution from the metal-optic resonator with the extended top metal plate as a function of the normalized pump power as well as the fitting curves with various *β* values based on the constant-temperature laser rate equations (*T* = 101 K). Due to the large mismatch between the gain peak and the resonance wavelength, the laser output intensity evolution is expected to have a large slope change near the threshold resulting from a relatively low *β* value (*β* ∼ 0.017). However, on the contrary, the measurement results in Fig. 4(a) show a smooth transition, typically observable with larger *β* values (∼0.1). We believe this discrepancy mainly comes from the temperature dependence of materials and resonator parameters. In the conventional semiconductor lasers, such temperature dependence in the lasing threshold, *β*, non-radiative recombination rate, and radiative recombination rate, has been often overlooked. However, in our situation, relatively low resonator Q factor requires large carrier density and strong electrical or optical pumping, resulting in significant temperature variation with respect to the input pump level^14^. Self-heating during the carrier injection process thus should be carefully taken into account to explain the measurement results. When we do not properly reflect the thermal effects in the rate equation model, the experimental results match closely with the case of *β* ∼ 0.1 although the actual resonance is quite different from the spontaneous emission peak (Fig. 3). The temperature-considered rate equation model (violet curves in Fig. 4) explains the experimental results well.

To consider the pump-dependent resonator temperature variation, we first obtained the relationship between the optical pump power and device temperature by solving thermal diffusion problems at each pump power condition using the finite element methods as shown in Fig. 1(h).


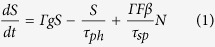



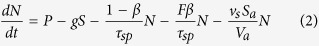


Temperature-dependent output intensity characteristics are then estimated through rate analysis of the lasing mode photon density *S* and the carrier density *N* as given by equation (1) and (2), respectively^25^, where *Γ* is the confinement factor, and *P* is a pump photon rate. The surface area of the semiconductor gain material *S*_*a*_ and the volume *V*_*a*_ are related with surface recombination. The optical gain, *g* = *cG(N-N*_*0*_*)/n*_*g*_, is assumed to be linear with respect to *N*, where *N*_*0*_*, c, G* and *n*_*g*_ represent the transparent carrier density, the light velocity, the differential gain coefficient, and the group refractive index, respectively. The photon life time, *τ*_*ph*_, is obtained from the cavity quality factor (*τ*_*ph*_ = *Q/*(*2πf*)). Temperature-dependent models for the transparent carrier density, *N*_*0*_^23^, the differential gain coefficient, *G*^26^, the SE lifetime, *τ*_*sp*_^27^, the photon life time, *τ*_*ph*_, and the surface recombination velocity, *v*_*s*_^28^ are considered. The SE coupling factor, *β*, is also closely related with temperature because of temperature-dependent semiconductor emission/gain spectra (peak wavelength shift and spectral broadening)^23^. The details of temperature-dependent *β* is explained in Figure S4. The temperature-dependent cavity resonance wavelength shift was not reflected in our analysis since the real part of the semiconductor material permittivity does not vary dramatically when compared to the SE characteristics. The relationship between the lasing threshold and temperature (*P*_*th*_ = *P*_*0*_exp(*T/T*_*0*_))^25^,^29^ was also taken into consideration with the estimated characteristic temperature of *T*_*0*_ = 75 K^30^. Further details are explained in the Supplementary material. In addition to the output intensity evolution curve from the temperature-dependent model (thicker violet curve), Fig. 4(b) also shows output intensity evolution curves (thin curves) at seven different temperatures assuming that the resonator temperature does not vary with the pump power. The actual evolution curve with temperature-dependent parameters can be quite different from the theoretical predictions with constant-temperature assumption.

## Conclusion

In summary, we design, fabricate, and characterize the subwavelength-scale metal-optic nanopatch resonators with the InGaAsP compound semiconductor cuboid structure sandwiched between two metal layers. The optical and thermal effects of such metallodielectric cavities were experimentally demonstrated and analyzed. Strong electromagnetic field confinement due to the metal layers allows enhanced spontaneous emission at resonances. Sufficient radiation Q factor enables the subwavelength-scale metal-optic nanopatch resonator with the extended top metal plate to reach a lasing condition under pulsed optical pumping at low temperature. It is also revealed that the effect of pump level-dependent temperature variation should be carefully taken into account in a laser rate equation model to better explain the experimentally observed output intensity evolution.

## Additional Information

**How to cite this article**: Kwon, K. *et al*. Optically pumped subwavelength-scale metallodielectric nanopatch resonators. *Sci. Rep.* **6**, 31793; doi: 10.1038/srep31793 (2016).

## Supplementary Material

Supplementary Information
