# Supplementary material for: Optically pumped subwavelength-scale metallodielectric nanopatch resonators
Source: Sci Rep. 2016 Aug 23;6:31793. doi: 10.1038/srep31793 (PMC4994098; doi:10.1038/srep31793)
Supplement: Supplementary Information [file srep31793-s1.pdf]

Supplementary Information

Optically pumped subwavelength-scale metallodielectric nanopatch resonators

*Kyungmok Kwon, Jong-bum You, Jaeho Shim, Youngho Jung, and Kyoungsik Yu**

Wheeler and McLean limit

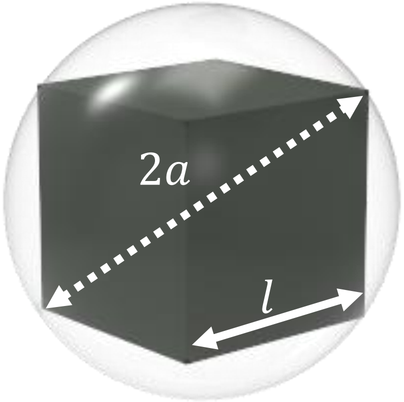

Figure S1. When a is the radius of the sphere enclosing the antenna structure, an electrically small antenna satisfies ka<1, where k is a wave vector ($2\pi/\lambda$), and λ is the free space wavelength. Under this condition, the minimum radiation Q factor from the McLean limit is $1/k^3a^3+1/ka$. For example, when the cube's side, l, is 350 nm, the radius of the sphere enclosing the cube is $a=\sqrt{3}l/2$~300 nm, which is close to the condition for the electrically small antenna's length scale.

Dielectric cavity and mode profile

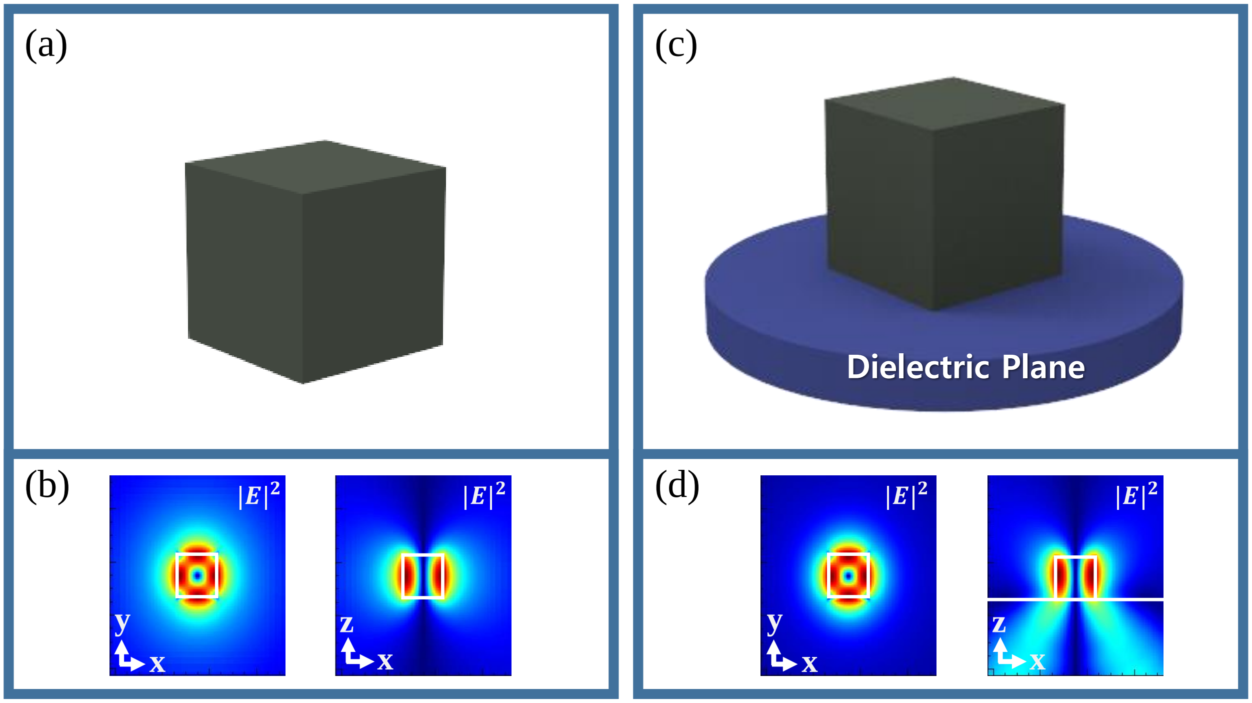

Figure S2. (a) Dielectric nano-cube block surrounded by air and (b) its TE-like mode profile. (c) Dielectric nano-cube block placed on the dielectric substrate and (d) its TE-like mode profile. The dielectric block shown in Figure S2(a) supports a very low Q factor of ~8.9 for the fundamental TE mode due to the diffraction limit and large radiation. When such a block is placed on the dielectric substrate, the optical energy can leak into the substrate, and the Q factor becomes even worse to ~3.2, approaching the Wheeler and McLean limit discussed in Figure S1. In our simulation, the cube block and the dielectric substrate plane were assumed as InGaAsP (n=3.57) and InP (n = 3.16), respectively. The side of the cube is assumed to be 350 nm. The resonant wavelengths for Figure S2(b) and Figure S2(d) are 1536 nm and 1496 nm, respectively.

Absorption of optical pump beam

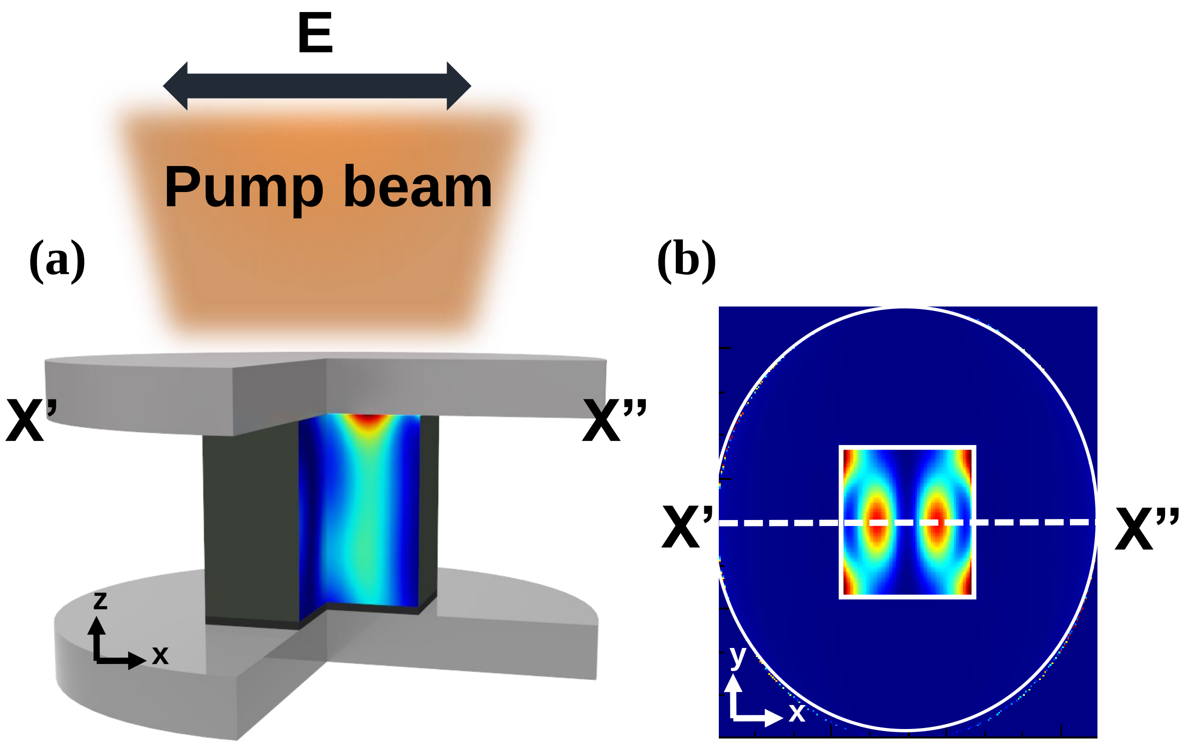

Figure S3. Input power absorption profile for a nanopatch resonator with an extended upper metal plate (d=1 μm) when the resonator is optically pumped from the surface normal direction using a 1064-nm laser. (a) Schematic of the metallodielectric nanopatch resonator. The color map shows the power absorption profile along the vertical cross-section of the semiconductor region. (b) Power absorption profile at the interface between top metal layer and semiconductor region. The absorption profiles indicate the optical pump energy is first converted to the surface plasmons on the metal surfaces, and then gets absorbed by the semiconductor cuboid. Due to the screening effects from the top metal layer, the optical pump energy transfer through direct free-space propagation is estimated to be negligible when d>l. Direct transmission of pump beam through the top metal layer is also negligible because of large optical losses associated with its thickness (>100 nm). The simulation results were obtained from three-dimensional finite-difference time-domain simulations (FDTD Solutions, Lumerical Solutions Inc.).

Temperature-dependent laser rate equation

To consider temperature dependence in the laser rate equations, temperature-dependent material parameters are carefully taken into consideration. The transparent carrier density, N_0,[1] and the differential gain coefficient, G,[2] were assumed linearly and inversely proportional to temperature, respectively. The spontaneous emission lifetime was estimated to be τ_{sp}~0.7 ns at 101K from the temperature dependent spontaneous emission coefficient, which is given by $B=5.69\text{x}10^{-12}\text{x}\ (exp(1413/(T+199.6)))$.[3] Only surface recombination was considered as a non-radiative recombination process because of the large surface-to-volume ratio and the cryogenic temperatures. At low temperatures, the effect of Auger recombination is negligible. We assume a surface recombination velocity of $v_s=1.6\times10^3\times\sqrt{T}\ \ cm/s$.[4] The photon life time, τ_{ph}, was obtained from the cavity quality factor ($\tau_{ph}=Q/(2\pi f)$), and the resonator quality factor Q also varies with the temperature. The temperature-dependent spontaneous emission coupling factor, β, was estimated from the temperature-dependent spectrum shift and spectral broadening of the semiconductor gain material. To consider the exponential temperature dependence of the lasing threshold pump power, relation between threshold and temperature ($P_{th}=P_0$ exp(T/T_0))[5,6] was taken into equation, and the characteristic temperature was estimated as T_0=75 K.[7]

Temperature-dependent spontaneous emission coupling factor

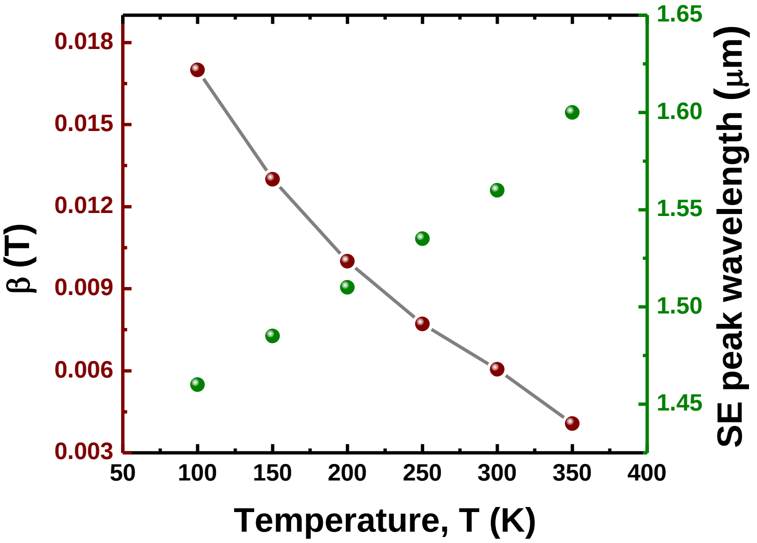

Figure S4. Spontaneous emission coupling factor, β, and peak wavelength of the spontaneous emission (SE) as a function of temperature for the lasing mode. The temperature dependence of β is obtained from $\beta(T) = \frac{R_{SP,lasing}(T)}{\sum_{mnp} R_{SP,mnp}(T)}$, where $R_{SP,lasing}$ is the rate of spontaneous emission into lasing mode and $R_{SP,mnp}$ is the rate of spontaneous emission into all modes.[1] The spontaneous emission red-shifts significantly with increasing temperature while the cavity resonant frequency is rarely varied near 1330 nm. As a result, β decreases with temperature.

Reference

1 Smalley, J. S. T., Gu, Q. & Fainman, Y. Temperature dependence of the spontaneous emission factor in subwavelength semiconductor lasers. *IEEE J. Quantum Electron.* **50**, 175-185, (2014).

2 Kim, C. K. & Lee, Y. H. Thermal characteristics of optical gain for GaInNAs quantum wells at 1.3 mu m. *Appl. Phys. Lett.* **79**, 3038-3040, (2001).

3 Zielinski, E., Schweizer, H., Streubel, K., Eisele, H. & Weimann, G. Excitonic transitions and exciton damping processes in InGaAs/InP. *J. Appl. Phys.* **59**, 2196-2204, (1986).

4 Forchel, A., Menschig, A., Maile, B. E., Leier, H. & Germann, R. Transport and optical-properties of semiconductor quantum wires. *J. Vac. Sci. Technol. B* **9**, 444-450, (1991).

5 Coldren, L. A., Corzine, S. W. & Mashanovitch, M. L. *Diode lasers and photonic integrated circuits*. Vol. 218 (John Wiley & Sons, 2012).

6 Lasher, G. & Stern, F. Spontaneous and stimulated recombination radiation in semiconductors. *Physical Review* **133**, A553, (1964).

7 Sugimura, A. Band-to-band Auger recombination in InGaAsP lasers. *Appl. Phys. Lett.* **39**, 21-23, (1981).
